# Supplementary material for: Engagement With Daily Testing Instead of Self-Isolating in Contacts of Confirmed Cases of SARS-CoV-2: A Qualitative Analysis
Source: Front Public Health. 2021 Aug 3;9:714041. doi: 10.3389/fpubh.2021.714041 (PMC8369371; doi:10.3389/fpubh.2021.714041)
Supplement: Supplementary file 1 [file Table_1.DOCX]

Supplement 1. Semi structured topic guide

Daily testing sample

1. What made you decide to carry out daily testing at home instead of quarantining?
   1. What were your feelings about quarantining?
   2. What were your feelings about daily testing?
2. What happened on the day you were told you had been in contact with someone with the virus?
   1. Can you tell me about your experiences on the day(s) that you were waiting for your test kit to arrive?

Experiences of testing

1. What happened on the first day that you were able to take a test? Did this change over time?
   1. When did your kits arrive?
   2. Were there any times that you didn’t test yourself?
   3. What happened on those days?
   4. What was the most difficult part of having to test yourself daily?
   5. What did you do to help you overcome any problems?

Behaviour during testing

1. What, if anything, did you do differently in the home during your seven days of daily testing?
   1. Did you take any extra precautions to reduce infection in the home?
   2. Why/why not?

Experiences of test results / impact on behaviour

1. How did you feel when you received a negative test result? How did it affect your life?
2. How did you feel when you received a positive test result? How did it affect your life?

**For those who were required to self-isolate**

1. What does the term self-isolation mean to you?
2. Can you tell me about your experiences of having to self-isolate?
   1. What steps did you take?
   2. What was the most difficult part of having to self-isolate?
   3. What did you do to overcome any problems you had?
   4. What would have helped you overcome any problems that you had?
   5. Do you think having to self-isolate had any impact on your health/wellbeing in anyway?

There’s loads of guidance to do with self-isolation, and we know people can find some of it tricky. Can you tell me about any times when you had to leave the house?

- 1. Can you tell me about any times when you had visitors?
  2. Can you tell me about any times it was hard to stick to the guidance?
  3. What, if anything, did you do differently in the home while you were self-isolating?

1. Did you take any extra precautions to reduce infection in the home?
   1. Why/why not?
   2. What support did you have to help you with daily testing and self-isolation?
   3. What did you think of the support?
   4. What support did you need? / what was missing?

Information

1. What information or advice did you have about daily testing?
   1. Where did you look for information or advice?
   2. What information did you find most reliable?
   3. What did you think about the information you had/found?
   4. Was anything unclear or confusing?
   5. Was anything missing?
2. What information or advice did you have about self-isolation?
   1. Where did you look for information or advice?
   2. What information did you find most reliable?
   3. What did you think about the information you had/found?
   4. Was anything unclear or confusing?
   5. Was anything missing?
3. Have you had to take a test or do quarantine before? How did your experiences of daily testing and self-isolation compare with any other times you have been in contact with a positive case?
   1. What was different?
   2. What was better/worse?
   3. If you were informed that you had been in contact with a positive case in the future, would you choose to complete seven days testing again or to quarantine instead?
   4. What might influence this decision?
   5. What could be done to make it better / easier for people to test/isolate?
4. If you had a positive test in the future and you knew that your contacts would be able to have daily testing (instead of self-isolating), would this affect how willing you are to share their contact details?
5. Is there anything else you would like to say?

Quarantining sample

1. Can you start off by telling me about your experiences of having to quarantine for 10/14 days?
   1. What made you decide to carry out quarantining instead of 7 days daily testing?
   2. Did you have any concerns about daily testing that made you choose quarantining?
   3. Did you have any concerns about quarantining?
   4. Were any of your family / household also a contact of the case?
   5. What happened on the day you were told you had been in contact with someone with the virus?
2. What does the term self-isolation mean to you?
   1. Can you tell me about your experiences of having to self-isolate?
   2. What steps did you take?
   3. Do you think having to self-isolate had any impact on your health/wellbeing in anyway? What was the most difficult part of having to quarantine?
   4. What did you do to help you overcome any problems?
   5. Did having to quarantine have any impact on your health in anyway?
3. There’s loads of guidance to do with self-isolation, and we know people can find some of it tricky. Can you tell me about any times when you had to leave the house?
   1. Can you tell me about any times when you had visitors?
   2. Can you tell me about any times it was hard to stick to the guidance?
   3. What would have helped you to be able to follow the advice around quarantining?
4. What, if anything, did you do differently in the home during the 10/14 days that you were in quarantine?
   1. Did you take any extra precautions to reduce infection in the home?
   2. Why/why not?
5. What information or advice did you have about quarantining?
   1. Where did you look for information or advice?
   2. What information did you find most reliable?
   3. What did you think about the information you had/found?
   4. Was anything unclear or confusing?
   5. Was anything missing?
   6. What support did you have to help you to quarantine?
   7. What did you think of the support?
   8. What support did you need? / what was missing?
6. If you were informed that you had been in contact with a positive case again, would you be willing to complete seven days testing / isolating instead of quarantining for 10/14 days?
   1. Why?
   2. What might influence this decision?
   3. What could be done to make it better / easier for people to test/isolate?
7. Is there anything else you would like to say?

Supplement 2: Analysis coding framework

| **Key theme** | **Code** | **Description/notes** |
| --- | --- | --- |
| **Factors influencing acceptance of testing** | | |
| **Protecting those around you** | Avoid putting others at risk | For participants who chose both daily testing and self-isolating, protecting themselves and those around them was a priority |
|  | Likelihood of transmitting the virus | Beliefs regarding the likelihood that the individual could have and could potentially spread the virus |
| **Need to avoid self-isolation** | Competing priorities | Including shopping, medication, exercise etc. Deliveries were not always available or feasible |
|  | Lack of need | Particularly among those who were able to work from home, had existing support networks and/or had strategies and supplies in place. |
| **Concerns about test sensitivity** | Concerns about the overall accuracy of LFD | Including a lack of understanding of how tests work and the stage at which the virus can be detected / transmitted. |
|  | Concerns about taking the test accurately |  |
| **Perceived benefits of detecting infection** | To know if I am infected | Being motivated to take tests in order to find out whether or not they had the virus. In particular, for those living in vulnerable households. |
| **Impact of test results** | | |
| **Positive consequences of confirmation of COVID status** | Confirmatory diagnosis | Tests provided confirmation of COVID status - particularly for those with atypical symptoms or symptoms that could be attributed to other conditions. |
|  | Benefits of rapid COVID diagnosis | Including mental wellbeing benefits associated with “knowing” COVID status |
| **Enabling essential activities** | Undertaking essential activities | Participants discussed appreciating being able to leave their homes for short period, for example if they had limited private or outdoor space, or had everyday tasks to complete |
|  | Impact of avoiding isolation on mental wellbeing | Participants discussed the impact of being able to leave the home on their mental health |
|  | Festive celebrations |  |
| **Uncertainty** | Understanding of rules and regulations | In particular, participants were confused about whether they could leave the home following a negative test result if they were living with a positive case |
|  | Novelty | The novelty of daily testing caused some concerns about the legitimacy of the approach, largely due to the contrast with the message to stay at home at all costs. |
| **Self isolating whilst testing** | Caution motivated over-adherence | Many participants gave reasons why they decided to self-isolate, even after receiving a negative LFD test |
|  | Spending time with household | Using the results of the test to facilitate within home contact, but not outside the home. |
